# A novel circular RNA circ_0020647 promotes ETEC-induced IPEC-J2 cell pyroptosis via the ssc-miR-185/BRD4 axis

**DOI:** 10.3389/fvets.2025.1578941

**Published:** 2025-06-04

**Authors:** Kaiqing Zhu, Puyu Liu, Kangping Liu, Yanan Cui, Pengxin Jiang, Xutao Wang, Ning Chen, Jiamei Cui, Zijuan Hou, Jianguo Li, Jinghui Fan, Yuzhu Zuo, Yan Li

**Affiliations:** ^1^College of Veterinary Medicine, Hebei Agricultural University, Baoding, China; ^2^College of Animal Science and Technology, Hebei Agricultural University, Baoding, China

**Keywords:** circ_0020647, ssc-miR-185, BRD4, ETEC, IPEC-J2, pyroptosis

## Abstract

**Introduction:**

Enterotoxigenic *E. coli* (ETEC) is a major pathogen causing piglet diarrhea. This study aimed to investigate the mechanism of porcine circular RNAs (circRNAs) in regulating intestinal immunity during ETEC infection.

**Methods:**

The circRNA expression profiles were obtained in ETEC-infected and uninfected IPEC-J2 cells via RNA-sequencing. The stable covalently closed structure of circRNAs was validated using qRT-PCR and RNase R digestion methods. The potential circRNA/miRNA/mRNA interactions were analyzed using Miranda software, dual-luciferase reporter assay, knockdown and over-expression of the target gene or RNA. The expression of pyroptosis-related factors was assessed by qRT-PCR and Western blot. Flow cytometry was utilized to quantify pyroptotic cells, and transmission electron microscopy was used to observe cellular morphology.

**Results:**

In this study, a total of 328 differentially expressed circRNAs were identified in ETEC-infected versus uninfected IPEC-J2 cells, among which a novel circRNA named circ_0020647 was significantly upregulated post-infection. Circ_0020647, encoded by an intergenic sequence, forms a covalently closed loop structure. We demonstrated that circ_0020647 acts as a molecular sponge for miRNA ssc-mir-185 through direct binding, which in turn targets BRD4 mRNA. Following ETEC infection, circ_0020647 promoted pyroptosis in IPEC-J2 cells by increasing the expression of NLRP3, GSDMD, and caspase-1. Additionally, circ_0020647 was involved in ETEC-induced cell injury, characterized by LDH efflux, IL-1β and IL-18 secretion, formation of membrane pores, and mitochondrial abnormalities. We revealed that the role of circ_0020647 in regulating pyroptosis was mediated by the ssc-mir-185/BRD4 axis.

**Conclusion:**

Our study constructed a novel circ_0020647/ssc-mir-185/BRD4 network that played an important role in the pyroptosis of IPEC-J2 cells induced by ETEC infection. Our findings imply that the circRNA/miRNA/mRNA network may be a novel biomarker and a potential therapeutic target for diarrhea in piglets caused by ETEC.

## 1 Introduction

Enterotoxigenic *Escherichia coli* (ETEC) is one of the most frequently identified pathogens causing bacterial diarrhea illness, including travelers’ diarrhea and children’s diarrhea in lower income-countries (1). It is also the major cause of diarrhea in piglets, resulting in great economic losses to the pig industry worldwide. ETEC colonizes the surface of the host intestinal epithelial cells via adhesins, followed by the release of heat-unstable enterotoxin (LT) or heat-stable enterotoxin (ST) (2). The enterotoxins further activate chloride channels in the intestinal epithelium, inducing the quantitative secretion of electrolytes and water, and ultimately leading to the occurrence of watery diarrhea, which is accompanied by gut immune responses (3, 4).

Many studies found that ETEC infection induced host intestinal inflammatory response and abnormal intestinal cell death. The upregulation of IL-1β, IL-18, and TNF-*α* was observed in ETEC K88 infected K88-infected IPEC-J2 cells (5). Consistently, the ETEC challenge also stimulated the production of inflammatory mediator IL-1β, IL-6, and TNF-*α* in the jejunum and IL-6 in the ileum of piglets (6). Epithelial cell death is one of the crucial steps in gut barrier integrity damage. It has been demonstrated that ETEC infection induces apoptosis of porcine small intestinal epithelial cells in piglets (7). Pyroptosis is a programmed cell death (PCD) that accompanies the inflammatory response and relies primarily on members of the Gasdermin protein family to form plasma membrane pores. It is often accompanied by morphological swelling of pyroptotic cells, perforation in the plasma membrane, release of pro-inflammatory factors, and eventually cell lysis (8, 9). A recent study reported that ETEC K88 induced pyroptosis of the IPEC-1 cell line via NLRP3/ GSDMD / caspase-1 pathway, indicating that pyroptosis might be a crucial step that links ETEC-induced intestinal immune responses and gut barrier physical integrity disruption (10). However, the regulatory mechanism of ETEC-induced pyroptosis in porcine intestinal cells remains unclear.

Circular RNAs (circRNAs) are a type of endogenous non-coding RNA, which is a closed loop structure connected by covalent bonds, participating in many potential biological functions by regulating gene transcription, acting as microRNA (miRNA) sponges, modulating protein–protein interaction, and directing peptide synthesis (11). As a miRNA sponge, circRNA could easily bind with miRNAs through complementary sequences to attenuate the inhibition of miRNA on its own target mRNAs, thereby increasing the expression level of miRNA target genes. This mechanism is known as the competitive endogenous RNA (ceRNA) mechanism. Numerous studies have reported that circRNAs are involved in muscle development, reproductive performance, and pathogenesis of infectious diseases via the ceRNA mechanism in domestic animals (12, 13). Li et al. established a ceRNA network of CircGLI3, miR-339-5p and VEGFA that regulates oxidative stress, thus affecting the proliferation of IPEC-J2 cells (14). Zhao et al. reported that porcine circ-SORBS1 sponges ssc-miR-345-3p to regulate its target gene SORBS1 expression, mediating cell adhesion to affect susceptibility to ETEC diarrhea of piglets (15). However, the regulatory mechanism of porcine circRNAs during ETEC-induced intestinal immunity and pyroptosis needs to be elucidated. In this study, we identified a novel circRNA, circ_0020647 in IPEC-J2 cells infected with ETEC F41, and constructed a competitive endogenous RNA network (ceRNA network) that regulates ETEC-induced pyroptosis of IPEC-J2 cells, providing potential targets for the diagnosis and treatment of *E. coli-infected* piglets’ diarrhea.

## 2 Materials and methods

### 2.1 Cell culture and ETEC infection

The ETEC F41 strain was purchased from the National Center for Veterinary Culture Collection (Beijing, China), and the IPEC-J2 cell and HEK293T cell line were purchased from Kobai Biological Company (Nanjing, China). IPEC-J2 cells were resuscitated in Dulbecco’s Modified Eagle’s medium (DMEM) (Gibco, United States) supplemented with 5% penicillin/streptomycin and 10% fetal bovine serum (FBS) (Gibco, United States). Cells were incubated at 37°C with 5% CO_2_ until 80% confluence, then DMEM was replaced with Eagle’s Minimal Essential Medium (opti-MEM) (Gibco, United States), followed by ETEC F41 infection at an MOI of 100 at 37°C for 1 h. After ETEC infection, the cell culture medium was discarded. Cells were rinsed 3 times with PBS, and incubated in hen cells cultured in DMEM containing 10% FBS for another 17 h. Finally, the cells were collected as ETEC-infected samples (18 h post-infection, 18 hpi). Uninfected IPEC-J2 cells were collected as control samples (0 hpi). Triplicates were set up for each group.

### 2.2 RNA extraction, library preparation and RNA-seq

Total cellular RNA was extracted with TRIzol (Invitrogen, United States), and RNA quality was examined using 1% agarose gel electrophoresis, spectrophotometer (IMPLEN, United States), and RNA 6000 Nano kit (Agilent Technologies, United States). The RNA samples (0 hpi and 18 hpi) were sent to Beijing Novozymes Technology Ltd. for library preparation and RNA-seq. First, ribosomal RNA was depleted by RibozeroTM rRNA Removal Kit (Epicenter, United States), and linear RNA was removed by RNase R (Epicentre, United States). Libraries were constructed by the NEBNext® UltraTM RNA Library Prep Kit (NEB, United States), followed by library quality assessment via the Agilent BioAnalyst 2,100 system (Agilent Technologies, United States). RNA-seq was performed on the Illumina PE150 platform (Illumina, United States).

### 2.3 Differentially expressed circRNAs (DEcircRNAs) screening and their parental gene enrichment analysis

The raw reads obtained from sequencing were filtered to remove low-quality reads. Reads with GC content >50%, Q20 ≤ 99% and Q30 ≤ 99.9% were selected as clean reads. Find_circ2 and CIRI2 were performed to analyze unbalanced junction reads and identify candidate circRNAs. Differential expression analysis of circRNAs was based on the negative binomial distribution of DESeq2. Log_2_(FoldChange) > 1 and adjusted *p*-value (*P*_adj_) < 0.05 was set as the threshold. Volcano plots and hierarchical clustering heatmaps were plotted for DEcircRNAs. The parental genes of DEcircRNAs were analyzed for Gene Ontology (GO) and Kyoto Encyclopedia of Genes and Genomes (KEGG) enrichment using GOseq and KOBAS 2.0 software, respectively.

### 2.4 qRT-PCR

The circRNA-specific primers were designed flanking the junction site (Supplementary Table 1) and synthesized by Sangon Biotech Co., Ltd. (Shanghai, China). Template cDNA was reverse transcribed from cellular total RNA using the HiScript® III RT SuperMix for qPCR (+gDNA wiper) kit (Vazyme, China), and qRT-PCR was performed using Taq Pro Universal SYBR qRT-PCR Master Mix (Vazyme, China) with *β*-actin as an internal reference gene. The cycling condition was as follows: Initial denaturation at 95°C for 10 min; 45 cycles of 95°C for 10 s and 60°C for 10 s; Melting curve cycle of 95°C for 10 s, 65°C for 1 min and 97°C for 1 s. The 2^−ΔΔCt^ method was utilized to analyze the relative changes in DEcircRNA expression in the ETEC-infected cells compared to the wild-type cells.

MicroRNA extraction was performed with RNAiso for Small RNA (Takara, Japan), followed by cDNA synthesis using the Mir-X™ miRNA FirstStrand Synthesis kit (Takara, Japan). Then, the Mir-X miRNA qRT-PCR TB Green Kit (Takara, Japan) was utilized to detect miRNA expression by PolyA plus tailing qPCR, in which U6 was used as the internal reference gene. The primers used are shown in Supplementary Table 2.

### 2.5 RNase R digestion

Total RNA was isolated from IPEC-J2 cells and divided into two halves. According to the manufacturer’s instructions, one half was subjected to RNase R digestion using the RNase R enzyme (Geneseed, China), while the other half was subjected to a control reaction using the same buffer without RNase R enzyme. Subsequently, treated RNA was reverse transcribed into cDNA for qRT-PCR using SYBR GreenI (Vazyme, China). The 2–ΔΔCt method was used to calculate the fold changes in circRNA expression normalized to *β*-actin.

### 2.6 Animal and tissue sampling

A total of six healthy 28-day-old Large White piglets that had been raised, under the same husbandry conditions were randomly selected. Euthanasia was carried out by exsanguination for tissue sampling, including kidney, spleen, lung, heart, liver, jejunum, ileum and duodenum. Collected samples were stored at-80°C after flash freezing in liquid nitrogen.

### 2.7 Cell transfection

The full-length circ_0020647 was synthesized by Sangon Biotech Co., Ltd. (Shanghai, China) and cloned into pCD25-ciR eukaryotic overexpression vector using EcoRI and BamHI restriction sites to generate a pCD25-ciR-circ_0020647 overexpression plasmid. IPEC-J2 cells were transfected with pCD25-ciR-circ_0020647 or pCD25-ciR empty vector as a negative control, respectively. The circ_0020647 siRNA (F: GCUCAAAGAAGUGGUUUUCTT; R: GAAAACCACUUCUUUGAGCTT), BRD4 siRNA (F: GGUGCACAUCAUUCAGUCUTT; R: AGACUGAAUGAUGUGCACCTT), ssc-miR-185 mimics, ssc-miR-185 inhibitor, and corresponding scrambled negative controls (NC) were synthesized by GenePharma (Suzhou, China). The IPEC-J2 cells that reached 50% confluence were transfected with plasmid or siRNA above using Lipofectamine 2000 (Thermo Fisher Scientific, United States) according to the manufacturer’s instructions. Transfection lasted for 6 h, then cells were cultured in DMEM containing 10% FBS for 30 h, followed by ETEC infection if required.

### 2.8 Dual luciferase assay

A circ_0020647 mutant and a BRD4 3’UTR mutant sequence were designed according to the ssc-miR-185 binding site, namely circ_0020647-MUT and BRD4-3’UTR-MUT, respectively. The circ_0020647-wild type (WT), circ_0020647-MUT, BRD4 3’UTR-WT, and BRD4-3’UTR-MUT were synthesized by Sangon Biotech Co. Ltd. (Shanghai, China) and constructed into the dual luciferase reporter vector pmirGLO (Promega, United States), respectively. The constructed dual luciferase reporter plasmids were named pmirGLO-circ_0020647-WT, pmirGLO-circ_0020647-MUT, pmirGLO-BRD4-3’UTR-WT, and pmirGLO-BRD4-3’UTR-MUT, respectively. HEK-293 T cells (ATCC, United States) were co-transfected with the above dual luciferase reporter plasmids and ssc-miR-185 mimics/NC using Lipofectamine 2000. Forty eight hours later, the activity of firefly luciferase and Renilla luciferase was determined using the Dual-Glo^®^Luciferase Assay System (Promega, United States) kit according to the manufacturer’s instructions.

### 2.9 Determination of cytoplasmic lactate dehydrogenase release

One milliliter of cell culture supernatant was collected for centrifugation at 12,000 × g for 20 min, subsequently, the supernatant was removed for LDH release determination using a Lactate Dehydrogenase Assay Kit (Njjcbio, China) according to the manufacturer’s instructions. The absorbance at 450 nm was measured using an ELx800 ELISA Microplate Reader (Bio-Tek, United States). LDH activity was calculated using the formula below: (U/LDH) = (A _assay_ − A _control_)/(A _standard_ − A _blank_) * C standard * N dilution * 1000, A _assay_, A _control_, A _standard_, and A _blank_ refer to the absorbance of the sample well, the absorbance of the control well, the absorbance of the standard well, and the absorbance of the blank well, respectively. C standard is the concentration of the standard substance, and N dilution is the dilution factor of the sample.

### 2.10 Measurement of inflammatory factors

The cell culture supernatant was collected for centrifugation at 1000 × g for 20 min, subsequently, the supernatant was tested for the secretion of the inflammatory factors IL-1β and IL-18 by Porcine interleukin 1β (IL-1β) enzyme-linked immunoassay (ELISA) kit (Mmbio, China) and Porcine interleukin 18 (IL-18) enzyme-linked immunoassay (ELISA) kit (Mmbio, China) according to the manufacturer’s instructions. The absorbance at 450 nm was measured using an ELx800 ELISA Microplate Reader (Bio-Tek, United States).

### 2.11 Transmission electron microscopy

Harvested cell samples were immediately pre-fixed in 2.5% glutaraldehyde (Solarbio, China) at 4°C overnight, then rinsed 3 times with PBS. After post-fixation in 1% osmium tetroxide for 2 h at room temperature, the samples were washed with PBS three times. The gradient alcohol solutions were applied for sample dehydration. Subsequently, infiltration was performed with acetone and Embed-812 resin mixture (v/v = 1/1) overnight, followed by embedding with pure Embed-812 resin overnight. Sections of 60–80 nm were cut with EM UC7 Ultra-thin Slicer (Leica, Germany), stained with dioscytium acetate and lead citrate, and observed using Transmission Electron Microscope HT7800 (HITACHI, Japan).

### 2.12 Flow cytometry

The cells were harvested, washed twice with PBS, and stained with the FAM-YVAD-FMK and PI using the Caspase 1 Live Cell Apoptosis Detection Kit (Life-lab, China). Data were obtained using a CytoFLEX Flow Cytometer (Beckman, United States) and analyzed by Kaluza Flow Cytometry Software.

### 2.13 Western blot assay

Total cellular protein was extracted using RIPA lysis buffer (RIPA: PMSF = 100:1) (Solarbio, China). Protein concentration in cell lysate was quantified using the BCA Protein Concentration Assay Kit (Solarbio, China). The denatured protein samples were separated on SDS-PAGE, followed by transfer to a PVDF membrane. The membrane was blocked in 5% milk in PBST for 1 h at room temperature. After primary antibody incubation at 4°C overnight, the membrane was incubated with a secondary antibody at room temperature for 1.5 h and proceeded with a DAB color development kit (Zsbio, China) for visualization. Quantification was achieved using ImageJ 1.8.0. The primary antibodies include NLRP3 (Proteinch, China, 1:1,000 dilution), caspase-1 (Proteinch, China, 1:1500 dilution), GSDMD (Affinity Biosciences, China,1:1,000 dilution), BRD4 (Affinity Biosciences, China, 1:1000 dilution), and *β*-actin (Abcam, UK, 1:2,000 dilution).

### 2.14 Statistical analysis

Each treatment was carried out in triplicate. Statistical analysis was performed using GraphPad Prism 8.0 software, and one-way ANOVA was used for significance analysis. A probability of *p* < 0.05 indicated statistical significance. *p ≤ 0.05, **p ≤ 0.01, ***p ≤ 0.001.

## 3 Results

### 3.1 CircRNA expression profile in IPEC-J2 cells infected with ETEC

**Figure 1:**
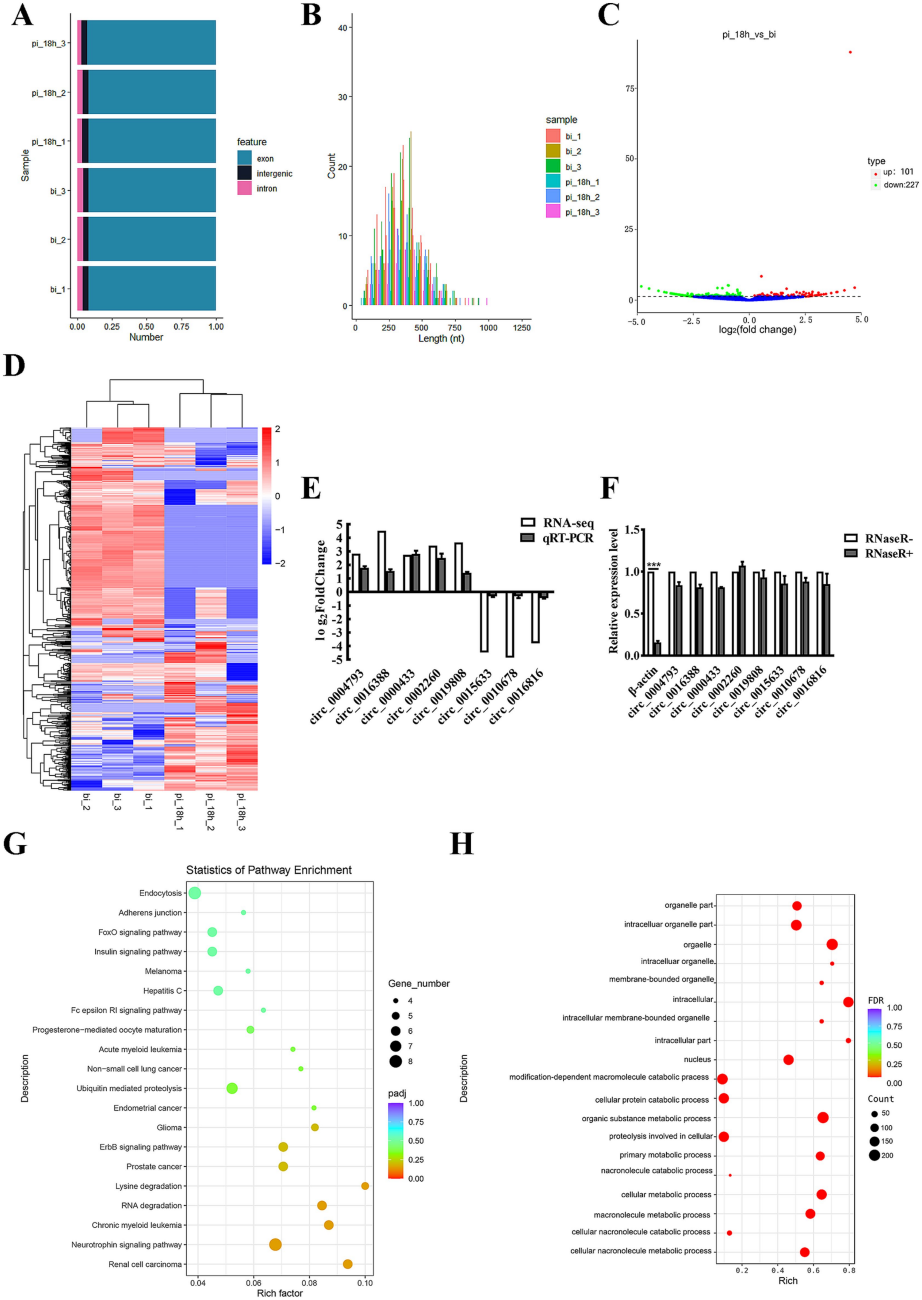
CircRNA expression profiles in IPEC-J2 cells before and after ETEC infection. **(A)** Genomic sources of circRNAs. The Y-axis displays sample name, pi_18h_1/pi_18h_2/pi_18h_3 indicate triplicates of IPEC-J2 cells at 18 h post-infection with ETEC, bi_1/bi_2/bi_3 indicate triplicates of uninfected IPEC-J2 cells. The X-axis shows the proportion of circRNAs derived from exon (blue), intergenic regions (black), and introns (pink). **(B)** Length distribution of circRNAs. **(C)** Volcano plot of DEcircRNAs in ETEC-infected IPEC-J2 cells versus uninfected cells. The red dots represent up-regulated DEcircRNAs (*p* < 0.05), the green dots represent down-regulated DEcircRNAs (*p* < 0.05), and the blue dots represent DEcircRNAs with no statistical difference (*p* > 0.05). **(D)** Clustering heatmap of DEcircRNA expression levels. Red and blue represent increased and decreased expression, respectively. **(E)** qRT-PCR validation of DEcircRNA expression. The y-axis displays the relative DEcircRNA expression which is represented with log_2_(Fold Change) comparing infected versus uninfected cells. **(F)** qRT-PCR validation of DEcircRNA digested by RNase R. β-actin was used as a linear RNA control. **(G)** KEGG signaling pathway analysis of the source genes of DEcircRNAs. **(H)** GO functional enrichment analysis of the source genes of DEcircRNAs. ***p ≤ 0.001.

A total of 77,413 potential circRNAs were identified in IPEC-J2 cells before (0 hpi) or post ETEC infection (18 hpi). The majority of circRNAs were between 250 and 500 nt in length, with the shortest and the longest being 33 nt and 1,262 nt, respectively (Figure 1A). 92.01% of the identified circRNAs originated from exonic regions, 4.02% were derived from introns, and another 3.97% were generated from intergenic segments (Figure 1B). Comparison of the porcine circRNAs in ETEC-infected IPEC-J2 cells with the control, a total of 328 differentially expressed circRNAs (DEcircRNAs) were discovered, of which 101 were upregulated and 227 were downregulated, indicating they might be associated with host-bacteria interaction. The expression profile of DEcircRNAs is visualized using a vacant plot and heat map (Figures 1C,D). The top 10 upregulated and downregulated DEcircRNAs in IPEC-J2 cells between 18 hpi and 0 hpi are listed in Table 1. The qRT-PCR validation suggested the expression trend of the randomly selected DEcircRNAs were similar to RNA-seq. (Figure 1E). Additionally, as shown in Figure 1F, circRNAs were more resistant to RNase R treatment compared to the control linear RNA, confirming that they contain stable circular structures. These results indicated that the circRNAs identified by RNA-seq in this study were reliable.

**Table 1:** The top 10 up-regulated and down-regulated DEcircRNAs in ETEC-infected IPEC-J2 cells versus uninfected cells.

CircRNA name	*P*_adj_	log_2_(Fold Change)	Regulation	Chromosome	Spliced length/bp	Annotation
circ_0020647	3.74E-05	4.7064	Up	AEMK02000292.1	234	Intergenic
circ_0016388	1.70E-88	4.5127	Up	6	65	Intergenic
circ_0001668	0.00032666	4.2472	Up	12	269	Exon
circ_0015583	0.0012274	3.9287	Up	5	135	Intergenic
circ_0016119	0.0013757	3.8964	Up	6	262	Exon
circ_0008563	0.0031055	3.6564	Up	1	461	Intron
circ_0019808	0.0031055	3.6564	Up	9	438	Exon
circ_0002260	0.0063304	3.4211	Up	12	251	Exon
circ_0016279	0.0063304	3.4211	Up	6	321	Exon
circ_0001674	0.008296	3.3236	Up	12	189	Exon
circ_0004719	3.52E-06	−5.0562	Down	14	779	Exon
circ_0010678	1.28E-05	−4.8246	Down	1	785	Exon
circ_0015633	7.64E-05	−4.4745	Down	6	358	Exon
circ_0003481	0.00050422	−4.056	Down	13	627	Exon
circ_0016816	0.0013947	−3.793	Down	6	589	Exon
circ_0009319	0.0019438	−3.7021	Down	1	519	Exon
circ_0002209	0.0038091	−3.5096	Down	12	405	Exon
circ_0018376	0.0045742	−3.4564	Down	8	376	Intergenic
circ_0019575	0.0045258	−3.4548	Down	9	623	Exon
circ_0015479	0.0046082	−3.4464	Down	5	422	Intergenic

To explore the potential biological functions of the porcine DEcircRNAs during ETEC infection, GO analysis was conducted. A total of 20 GO terms with significant enrichment (*P*_adj_ < 0.05) were detected, such as bioadhesion, immune system processes, cell membrane, organelle, extracellular matrix and synapse (Figure 1H). The top 20 enriched KEGG categories are presented in Figure 1G, including RNA degradation, lysine degradation, prostate cancer, ubiquitin-mediated proteolysis, adherens junction, endocytosis and so on. We suppose the pathways of various types of cancers might be associated with host cell proliferation and programmed cell death. Hepatitis C, adhesion junctions, and endocytosis are related to host-pathogen interaction and disease pathogenesis.

### 3.2 Identification and validation of a novel DEcircRNA circ_0020647

**Figure 2:**
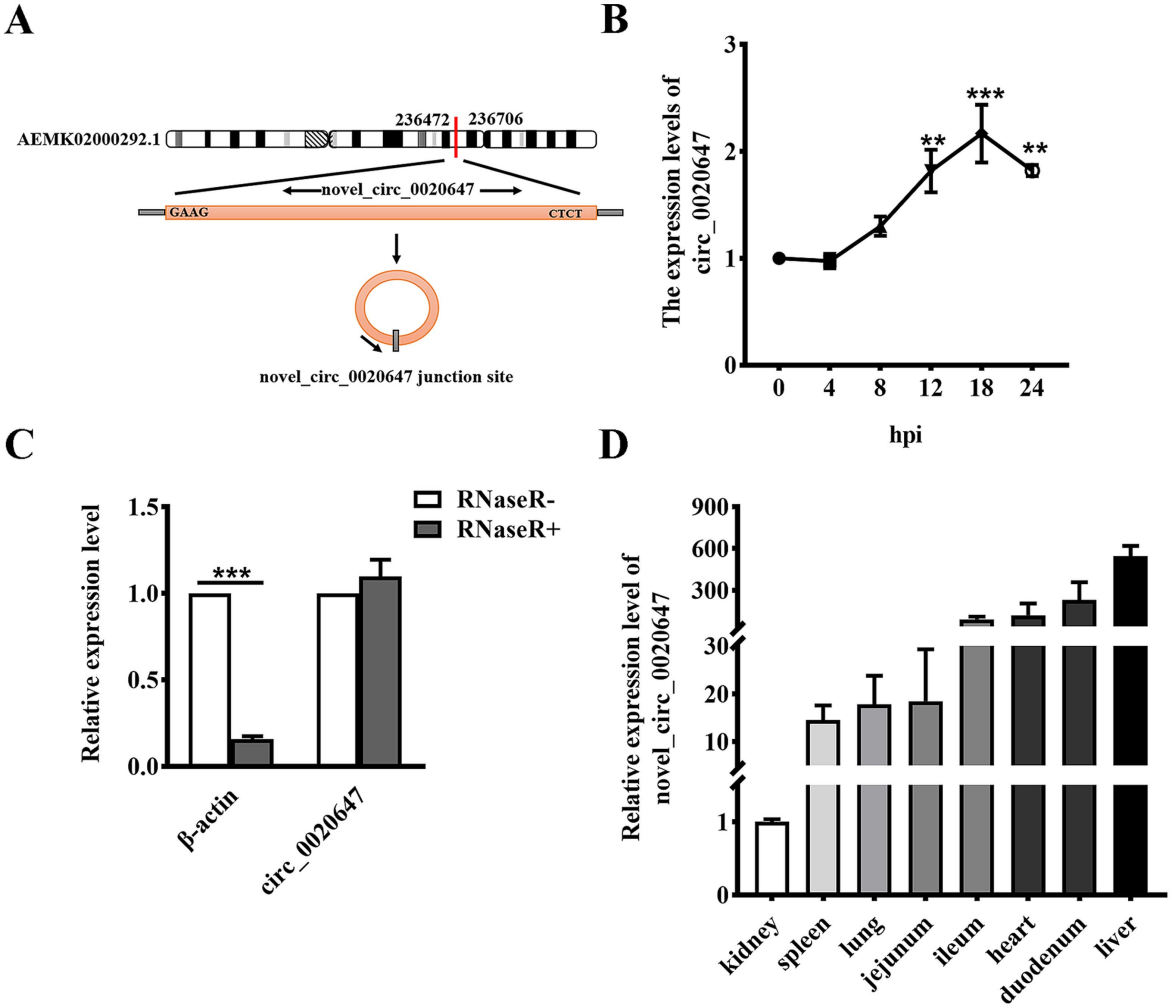
Identification of novel porcine circRNA circ_0020647. **(A)** circ_0020647 location and cyclization in the genome. The red line indicates the junction site of circ_0020647. **(B)** Relative expression of circ_0020647 at different time points of ETEC infection. The X-axis indicates 0, 4, 8, 12, 18, 24 h post-infection (hpi). The Y-axis shows the relative expression of circ_0020647 normalized to that at 0 hpi. **(C)** qRT-PCR validation of circ_0020647 digested by RNase R. β-actin was used as a linear RNA control. **(D)** Relative expression abundance of circ_0020647 in various tissues of piglets. The y-axis shows the relative expression of circ_0020647 in different tissues normalized to that in the kidney. **p ≤ 0.01, ***p≤ 0.001.

The current study focused on one of the novel DEcircRNAs, namely circ_0020647, the expression of which was significantly upregulated in IPEC-J2 cells post ETEC infection with a log_2_(Fold Change) of 4.7064. Circ_0020647 is encoded by an intergenic sequence on *sus scrofa* scaffold ID AEMK02000292.1 (Figure 2A). As shown in Figure 2B, the relative expression of circ_0020647 in IPEC-J2 cells gradually increased during ETEC infection, until it reached the highest level at 18 hpi, suggesting expression of endogenous circ_0020647 is probably relevant to host-ETEC interaction. RNase R treatments, combining qRT-PCR, verified that circ_0020647 displayed a stable covalently closed structure (Figure 2C). We also examined the distribution of circ_0020647 in different porcine tissues using qRT-PCR, and the highest abundance of circ_0020647 expression was found in the duodenum and liver (Figure 2D).

### 3.3 Circ_0020647 acts as a sponge of ssc-miR-185

**Figure 3:**
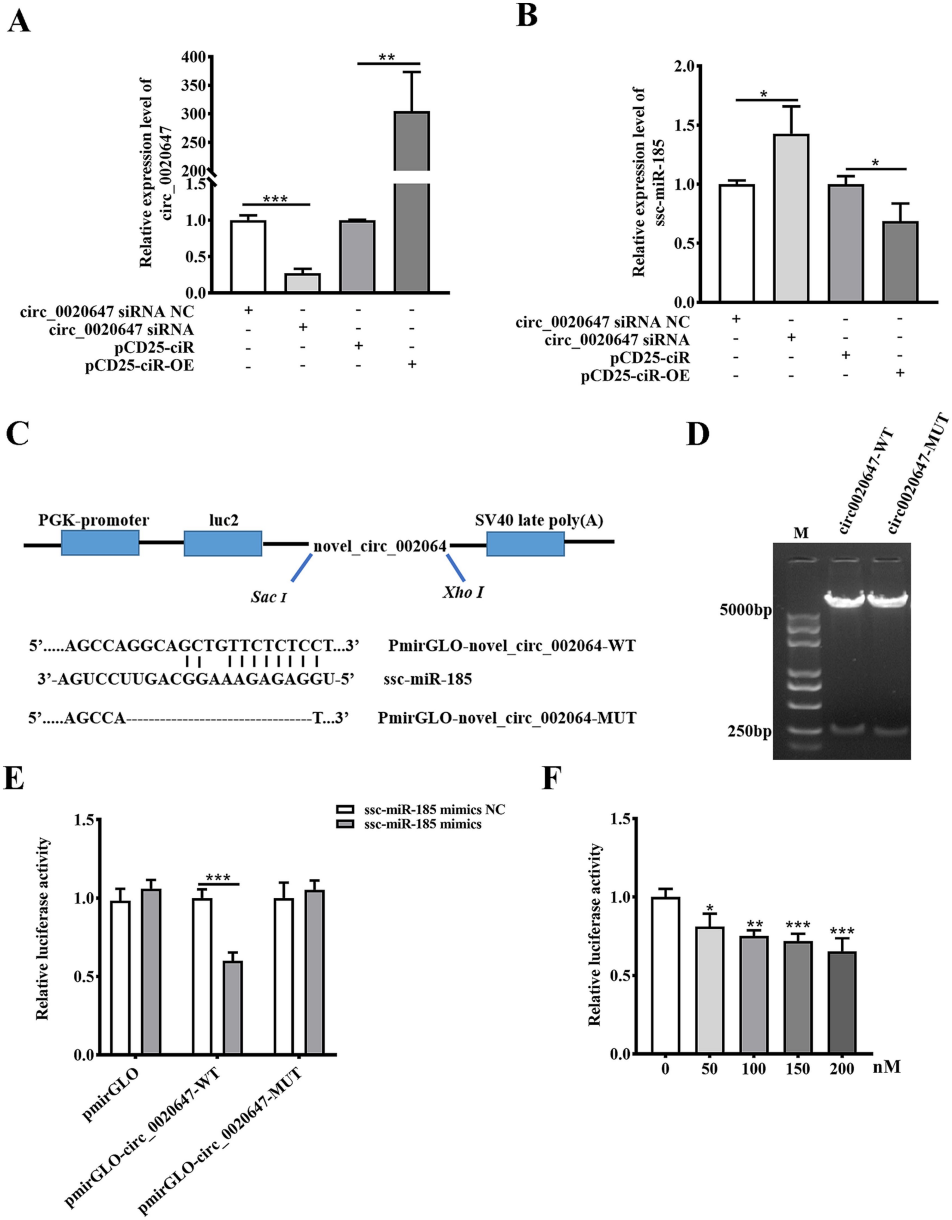
Circ_0020647 absorbs ssc-miR-185 by directly binding. **(A)** Knock-down and overexpression of circ_0020647. IPEC-J2 cells were transfected with circ_0020647 siRNA NC (scrambled control), circ_0020647 siRNA, pCD25-ciR (empty vector), or Pcd25-ciR-OE (pCD25-ciR-circ_0020647 overexpression plasmid). **(B)** Relative expression level of ssc-miR-185 in circ_0020647 KD and over-expression in IPEC-J2 cells. **(C)** Schematic diagram of PmirGLO-circ_0020647-WT/MUT plasmid construction and the prediction of the binding sites between circ_0020647 and ssc-miR-185. Circ_0020647-WT/MUT sequences were inserted at the 3’UTR of the firefly luciferase gene in the PmirGLO vector, respectively. **(D)** Electrophoresis of PmirGLO-circ_0020647-WT (lane 1) and PmirGLO-circ_0020647-MUT (lane 2) plasmids that double double-digested by SacI and XhoI. **(E)** Dual luciferase activity assay to detect the binding between circ_0020647 and ssc-miR-185. The Y-axis displays relative luciferase activity normalized to that of the HEK293 cells transfected with empty pmirGLO vectors and ssc-miR-185 mimics NC. The X-axis shows the treatments of the HEK293T cells, transfected with pmirGLO empty vector, pmirGLO-circ_0020647-WT or pmirGLO-circ_0020647-MUT. The bars in white indicate the cells transfected with ssc-miR-185 mimics control (ssc-miR-185 mimics NC), while the grey bars indicate the cells treated with ssc-miR-185 mimics. **(F)** Dual luciferase activity assay of HEK293T cells containing pmirGLO-circ_0020647-WT transfected with a gradient amount of ssc-miR-185 mimics. The Y-axis displays relative luciferase activity normalized to that of the HKE293T cells transfected with 0 nM ssc-miR-185 mimics. The X-axis indicates the cells were transfected with 0, 50, 100, 150, 200 nM of ssc-miR-185 mimics. *p ≤ 0.05, **p ≤ 0.01, ***p ≤ 0.001.

Based on the Miranda 3.3 software prediction, circ_0020647 contained complementary binding of a porcine microRNA named ssc-miR-185. To characterize the sponge action of circ_0020647 on ssc-miR-185, we knocked down circ_0020647 by siRNA and overexpressed circ_0020647 by plasmid construction in IPEC-J2 cells, respectively. The results suggested that in IPEC-J2 cells, up-regulating circ_0020647 resulted in a significantly declined level of native ssc-miR-185, while down-regulating circ_0020647 increased the level of ssc-miR-185 (Figures 3A,B). To confirm the direct binding of ssc-miR-185 with circ_0020647, the dual luciferase assay was conducted by cloning the circ_0020647 wild-type sequence (circ_0020647-WT) or mutated sequence (circ_0020647-MUT) into the pmirGLO vector as the 3’UTR of the firefly luciferase gene (Figures 3C,D). Using the circ_0020647-WT sequence as the 3’UTR, ssc-miR-185 mimics significantly reduced the luciferase signal. When circ_0020647-MUT was cloned into the 3’ UTR of the luciferase gene, there was no significant difference in signals between cells transfected ssc-miR-185 mimics or its scrambled control, indicating ssc-miR-185 targets the complementary sequence of circ_0020647 (Figure 3E). In addition, ssc-miR-185 mimics reduced the luciferase signal of the firefly luciferase gene with circ_0020647-WT sequence at 3’UTR in a dose-dependent manner, demonstrating there was a direct binding between ssc-miR185 and circ_0020647 (Figure 3F). Collectively, these results showed that porcine circ_0020647 acts as a sponge of ssc-miR-185.

### 3.4 Circ_0020647 promotes ETEC-induced inflammation via absorbing ssc-miR-185

**Figure 4:**
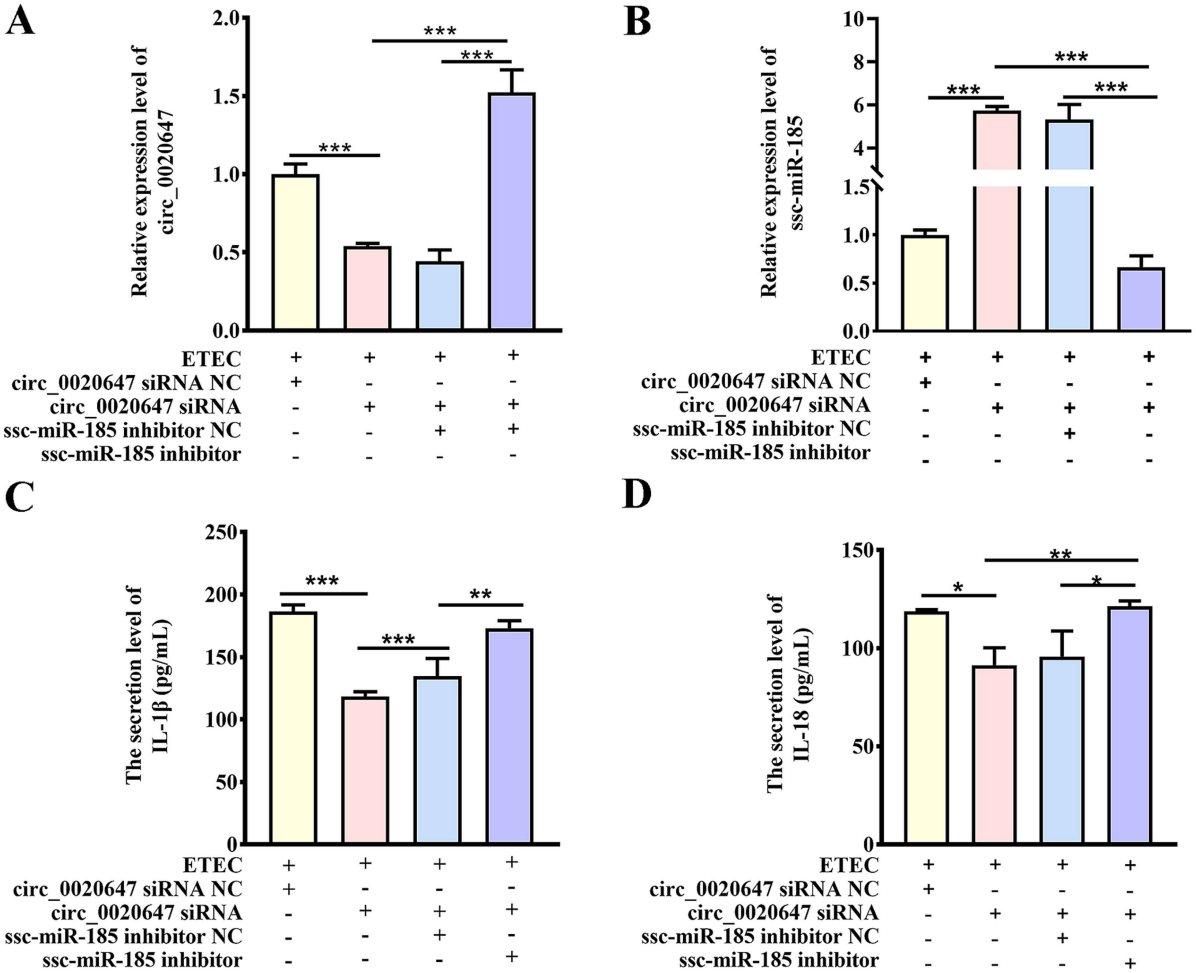
Circ_0020647 promotes ETEC-induced inflammation of IPEC-J2 cells via absorbing ssc-miR-185. **(A)** Relative expression of circ_0020647. The Y-axis shows the relative expression of circ_0020647 normalized to that of IPEC-J2 cells transfected with circ_0020647 siRNA NC (scrambled control) post ETEC infection. The X-axis represents treatments of the cells, including ETEC infection, transfection with circ_0020647 siRNA NC, circ_0020647 siRNA, ssc-miR-185 inhibitor NC (scrambled control), or ssc-miR-185 inhibitor. **(B)** Relative expression of ssc-miR-185. **(C)** IL-1β secretion of IPEC-J2 cells. **(D)** IL-18 secretion of IPEC-J2 cells. *p ≤ 0.05, **p ≤ 0.01, ***p≤ 0.001.

We further investigated whether the circ_0020647/ssc-miR-185 regulatory pathway affected the host immune response during ETEC infection. We knocked down circ_0020647 in IPEC-J2 cells using circ_0020647 siRNA, or double knocked down circ_0020647 and ssc-miR-185 via co-transfection of circ_0020647 siRNA and ssc-miR-185 inhibitors (double KD), followed by confirmation of their expression levels using qRT-PCR (Figures 4A,B). The secretion levels of inflammatory factors were tested using ELISA post-ETEC infection. Compared to the negative control (NC) cells, the secretion of IL-1β and IL-18 was significantly down-regulated when circ_0020647 was knocked down, suggesting that circ_0020647 promoted inflammation stimulation. While the addition of ssc-miR-185 inhibitor diminished the inflammation attenuation by circ_0020647 siRNA, confirming that ssc-miR-185 mediated the inflammatory responses promoted by circ_0020647 in ETEC-infected IPEC-J2 cells (Figures 4C,D).

### 3.5 Circ_0020647/ssc-miR-185 regulatory network promotes pyroptosis of ETEC-infected IPEC-J2 cells

**Figure 5:**
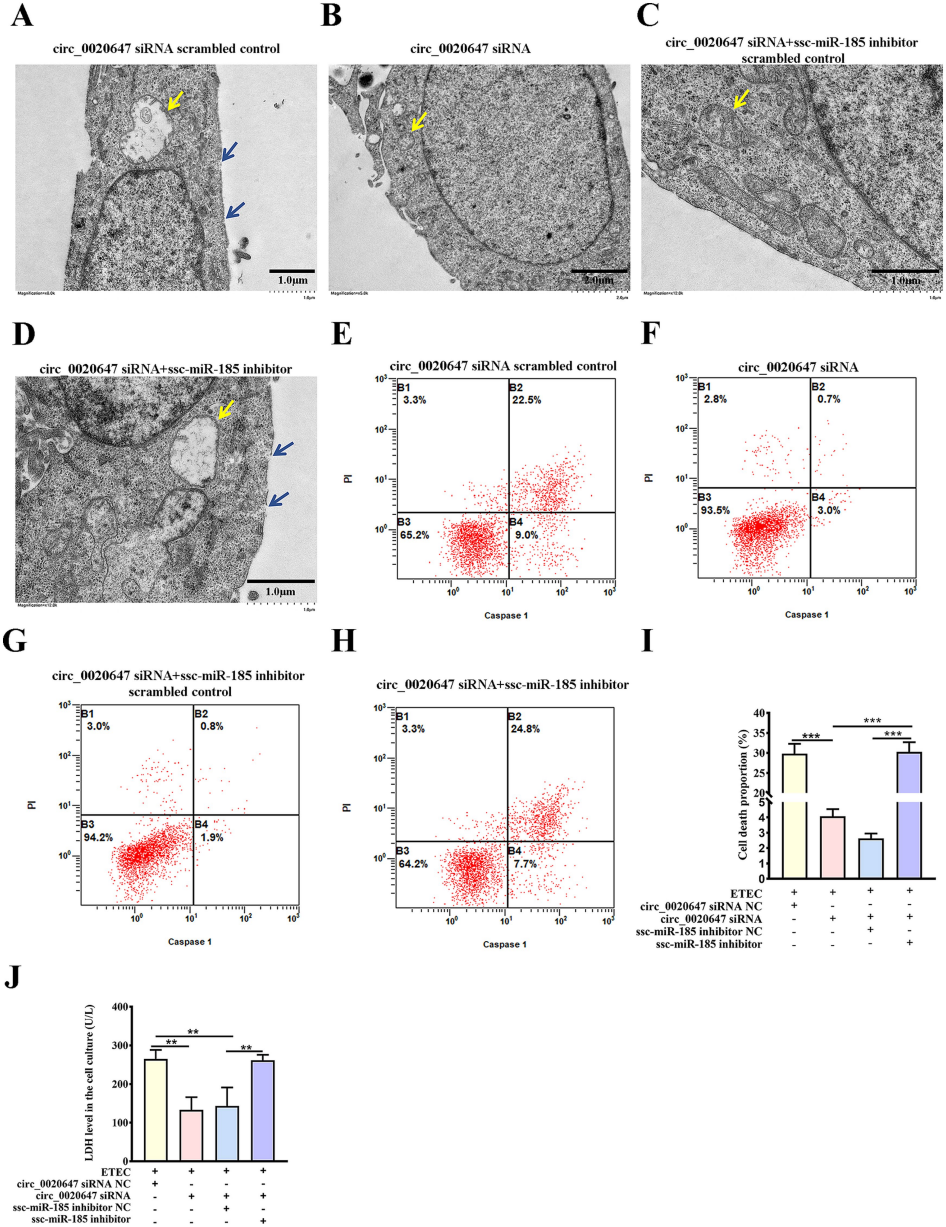
Ssc-miR-185 mediates circ_0020647 to promote pyroptotic cell death induced by ETEC infection. TEM of ETEC-infected IPEC-J2 cells transfected with circ_0020647 siRNA scrambled control **(A),** circ_0020647 siRNA **(B)**, circ_0020647 siRNA+ssc-miR-185 inhibitor scrambled control **(C),** circ_0020647 siRNA+ssc-miR-185 inhibitor **(D)**. The blue arrows point to the membrane pores. The yellow arrows point to the mitochondria. Cell death mediated by Caspase-1 was detected by flow cytometry in IPEC-J2 cells transfected with circ_0020647 siRNA scrambled control **(E)**, circ_0020647 siRNA **(F)**, circ_0020647 siRNA+ssc-miR-185 inhibitor scrambled control **(G)**, circ_0020647 siRNA+ssc-miR-185 inhibitor **(H)** post ETEC infection. **(I)** The cell death proportions in flow cytometry were quantified. **(J)** LDH release of ETEC infected IPEC-J2 cells. **p ≤ 0.01, ***p ≤ 0.001.

**Figure 6:**
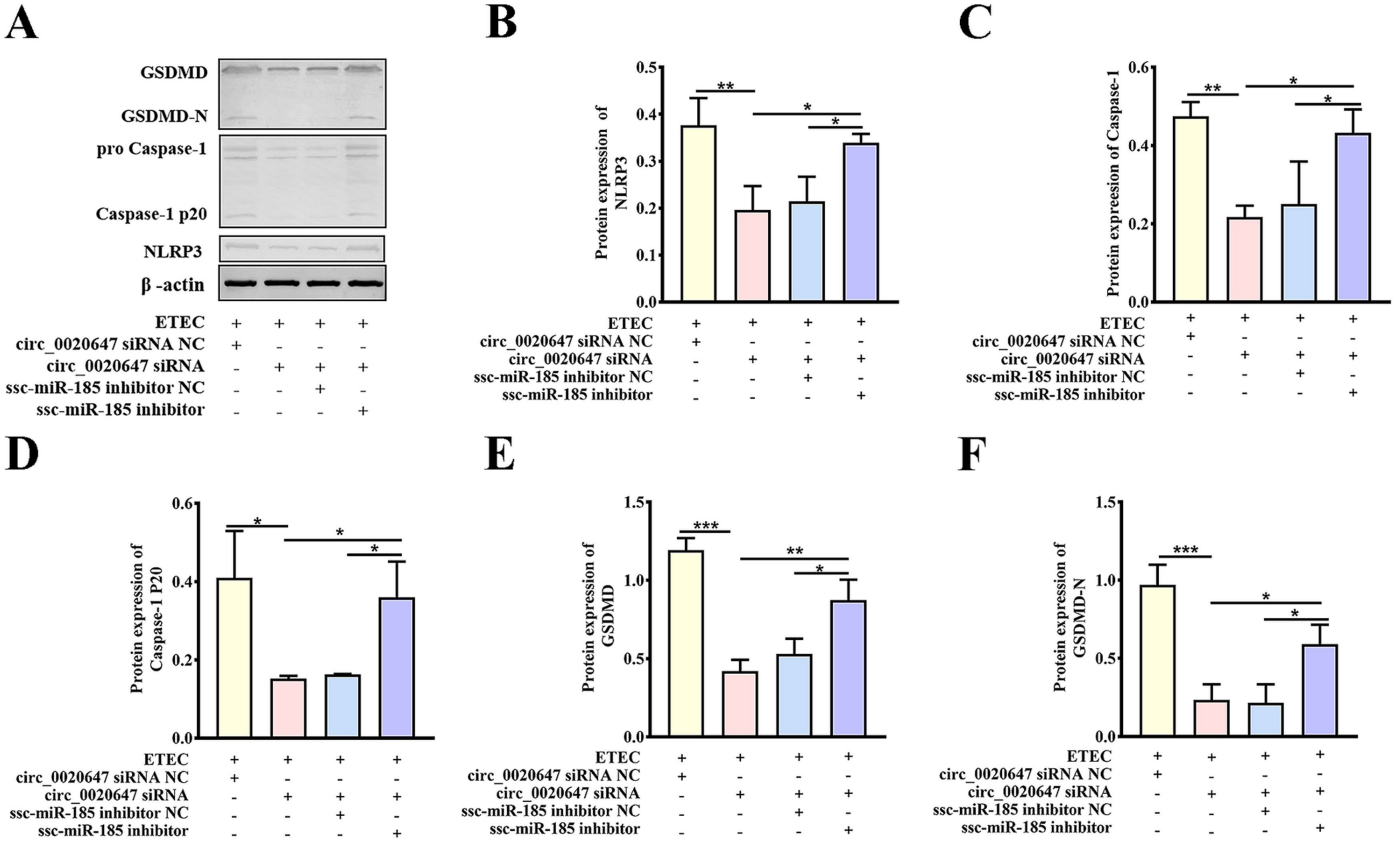
Biochemical characterization of pyroptotic indicators in circ_0020647/ssc-miR-185 KD cells. **(A)** Expressions of pyroptosis-related proteins were detected in cells with the indicated treatments by Western blot. ETEC infected IPEC-J2 cells were transfected with circ_0020647 siRNA scrambled control, circ_0020647 siRNA, circ_0020647 siRNA+ssc-miR-185 inhibitor scrambled control, circ_0020647 siRNA+ssc-miR-185 inhibitor. The relative protein levels of NLRP3 **(B)**, Caspase-1 **(C)**, Caspase-1 P20 **(D)**, GSDMD **(E)**, GSDMD-N **(F)** were further quantitatively analyzed by ImageJ. *p ≤ 0.05, **p ≤ 0.01, ***p ≤ 0.001.

To further explore the function of the circ_0020647/ssc-miR-185 network in IPEC-J2 cells induced by ETEC infection, we observed cell morphology changes using TEM. Characteristic plasma membrane perforation, mitochondrial swelling and vacuolization were noted in the wild-type IPEC-J2 cells post ETEC infection, while circ_0020647 KD cells appeared nearly intact cell membranes and slight mitochondrial damage, indicating circ_0020647 is involved in the activation of ETEC-induced pyroptosis (Figures 5A,B). Meanwhile, ssc-miR-185 inhibitor-transfected circ_0020647 KD cells showed typical pyroptotic morphologies again, demonstrating that circ_0020647 sponged ssc-miR-185 to improve ETEC-induced pyroptosis (Figures 5C,D). Flow cytometry analysis demonstrated that the proportion of caspase-1-mediated cell death induced by ETEC infection was remarkably decreased after the knockdown of circ_0020647, but the addition of ssc-miR-185 inhibitor induced a higher rate of cell death (Figures 5E–I). It indicates that circ_0020647 knockdown abolished its role as ssc-miR-185 sponge, leading to an increase in ssc-miR-185 level, which is associated with pyroptotic cell death. Robust cytoplasmic protein LDH efflux was detected from wild-type control cells exposed to ETEC, indicating ETEC infection increased cell permeability. Knockdown of circ_0020647 blocked LDH release, while double KD of circ_0020647 and ssc-miR-185 induced cell lysis again (Figure 5J). Consistently, both the mRNA and protein levels of pyroptosis mediators Gasdermin D (GSDMD), NLRP3 and Caspase-1 mRNA were obviously lower in circ_0020647 KD cells than those in the wild-type cells after exposure to ETEC (Supplementary Figures 1A–C; Figures 6A–F). Whereas, their expression displayed significant upregulation when cells were co-transfected with ssc-miR-185 inhibitors and circ_0020647 siRNA. As shown in Figures 6A,D, cleavage of caspase-1 happened with a clear detection of subunit p20 in both wild-type IPEC-J2 cells and double KD cells, suggesting caspase-1-mediated pyroptosis was activated. Finally, taken together, these results suggested that pyroptosis of ETEC-infected IPEC-J2 cells was promoted by circ_0020647 via absorbing ssc-miR-185.

### 3.6 Circ_0020647/ssc-miR-185 axis targets and regulates BRD4 expression during ETEC infection

**Figure 7:**
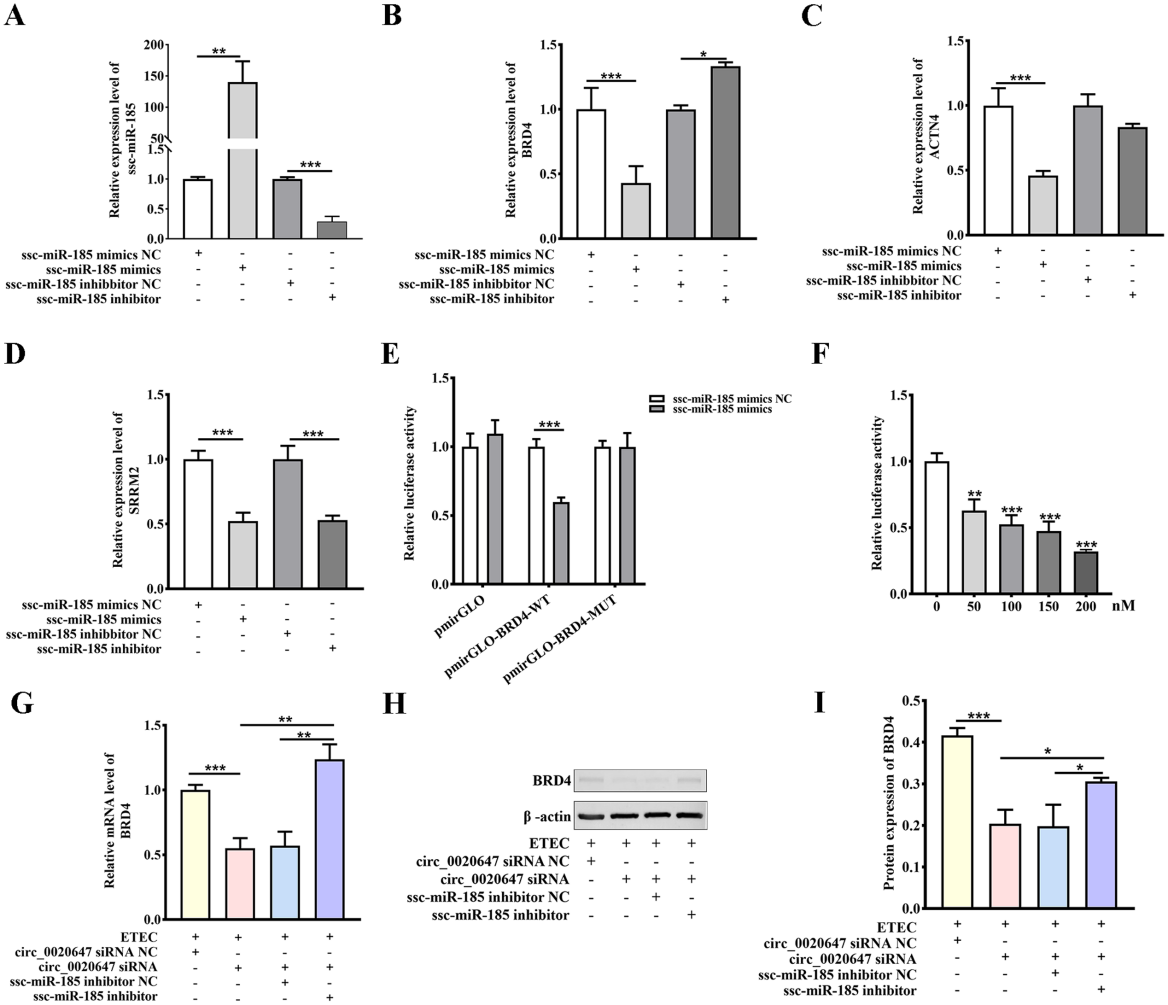
Ssc-miR-185 regulates BRD4 expression by direct binding. **(A)** Overexpression and inhibition of ssc-miR-185. IPEC-J2 cells were transfected with ssc-miR-185 mimics NC (scrambled control), ssc-miR-185 mimics, ssc-miR-185 inhibitor NC (scrambled control), and ssc-miR-185 inhibitor. The Y-axis displays the relative RNA level of ssc-miR-185 normalized to that in cells transfected with ssc-miR-185 mimics NC. Relative mRNA levels of BRD4 **(B)**, ACTN4 **(C)**, SRRM2 **(D)** detected by qRT-PCR were normalized to those in cells transfected with ssc-miR-185 mimics NC. **(E)** Dual luciferase activity assay to detect the binding between BRD4 and ssc-miR-185. The Y-axis displays relative luciferase activity normalized to that of the HEK293 cells transfected with empty pmirGLO vectors and ssc-miR-185 mimics NC. The X-axis shows the treatments of the HEK293T cells, transfected with pmirGLO empty vector, pmirGLO-BRD4-WT or pmirGLO-BRD4-MUT. The bars in white indicate the cells were transfected with ssc-miR-185 mimics control, while the grey bars indicate the cells were treated with ssc-miR-185 mimics. **(F)** Dual luciferase activity of HEK293T cells containing pmirGLO-BRD4-WT transfecting with a gradient amount of ssc-miR-185 mimics. The Y-axis displays relative luciferase activity normalized to that of the HKE293T cells transfected with 0 nM ssc-miR-185 mimics. The X-axis indicates the cells were transfected with 0, 50, 100, 150, 200 nM of ssc-miR-185 mimics. **(G)** Relative mRNA level of BRD4 in ETEC-infected IPEC-J2 cells transfected with circ_0020647 siRNA NC(0), circ_0020647 siRNA, circ_0020647 siRNA+ssc-miR-185 inhibitor NC, circ_0020647 siRNA+ssc-miR-185 inhibitor. **(H)** Western blot detection of BRD4 protein levels **(I)** Quantitative analysis of Western blot. *p ≤ 0.05, **p ≤ 0.01, ***p ≤ 0.001.

Three target genes of ssc-miR-185, including SRRM2, ACTN4 and BRD4, were predicted using Miranda d software. IPEC-J2 cells were transfected with ssc-miR-185 mimics or inhibitors, respectively (Figure 7A). Only the mRNA expression level of endogenous BRD4 was negatively correlated with ssc-miR-185 level (Figure 7B), but neither ACTN4 nor SRRM2 (Figures 7C,D). It indicates that BRD4 was the potential target of ssc-miR-185. To further verify the direct binding between ssc-miR-185 and BRD4 3’UTR sequence, a dual luciferase assay was performed in HEK293T cells transfecting ssc-miR-185 mimics, PmirGLO plasmids cloned with either WT BRD4 3’UTR (PmirGLO-BRD4-WT) or a mutated sequence at the ssc-miR-185 binding site (PmirGLO-BRD4-MUT) (Supplementary Figure 2). The results revealed that ssc-miR-185 mimics inhibited the luciferase signal of HEK293T cells containing the PmirGLO-BRD4-WT, but not that of the cells containing PmirGLO-BRD4-MUT (Figure 7E). Meantime, transfecting ssc-miR-185 mimics inhibited the luciferase signal of the cells containing PmirGLO- BRD4-WT sequence in a dose-dependent manner (Figure 7F), indicating that ssc-miR-185 directly targets the 3’UTR of BRD4 mRNA. Furthermore, after exposure to ETEC, a significant decrease in BRD4 expression was detected in circ_0020647 KD cells compared with the control, whereas simultaneous transfection of ssc-miR-185 inhibitor significantly upregulated BRD4 expression, demonstrating that circ_0020647 positively regulated BRD4 expression via sponging ssc-miR-185in IPEC-J2 cells during ETEC infection (Figures 7G–I). In summary, we constructed a circ_0020647/ ssc-miR-185/BRD4 ceRNA network, which is involved in host-ETEC interaction.

### 3.7 BRD4 regulates ETEC-induced pyroptosis of IPEC-J2 cells

**Figure 8:**
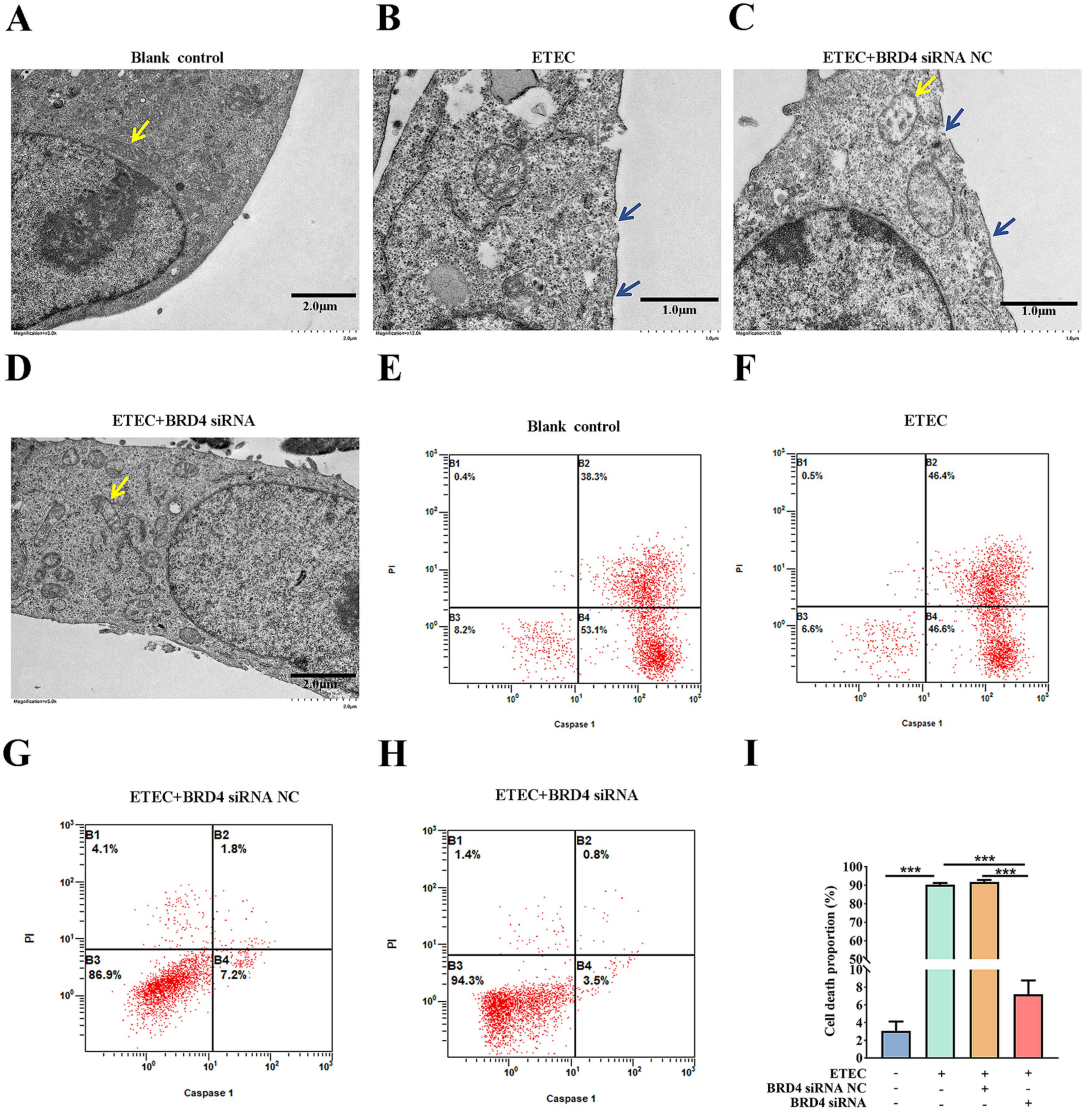
BRD4 promotes ETEC infection-induced pyroptosis of IPEC-J2 cells. TEM characterization of wildtype IPEC-J2 cells **(A)**, ETEC-infected IPEC-J2 cells **(B)**, ETEC-infected IPEC-J2 cells transfected with BRD4 siRNA NC (scrambled control) **(C)**, and ETEC-infected IPEC-J2 cells transfected with BRD4 siRNA **(D)**. The yellow arrows point to the mitochondria. The blue arrows indicate plasma membrane perforation. **(E–H)** Flow cytometry was performed to detect cell death of IPEC-J2 cells with the above treatments. **(I)** The cell death proportions in flow cytometry were quantified. ***p ≤ 0.001.

**Figure 9:**
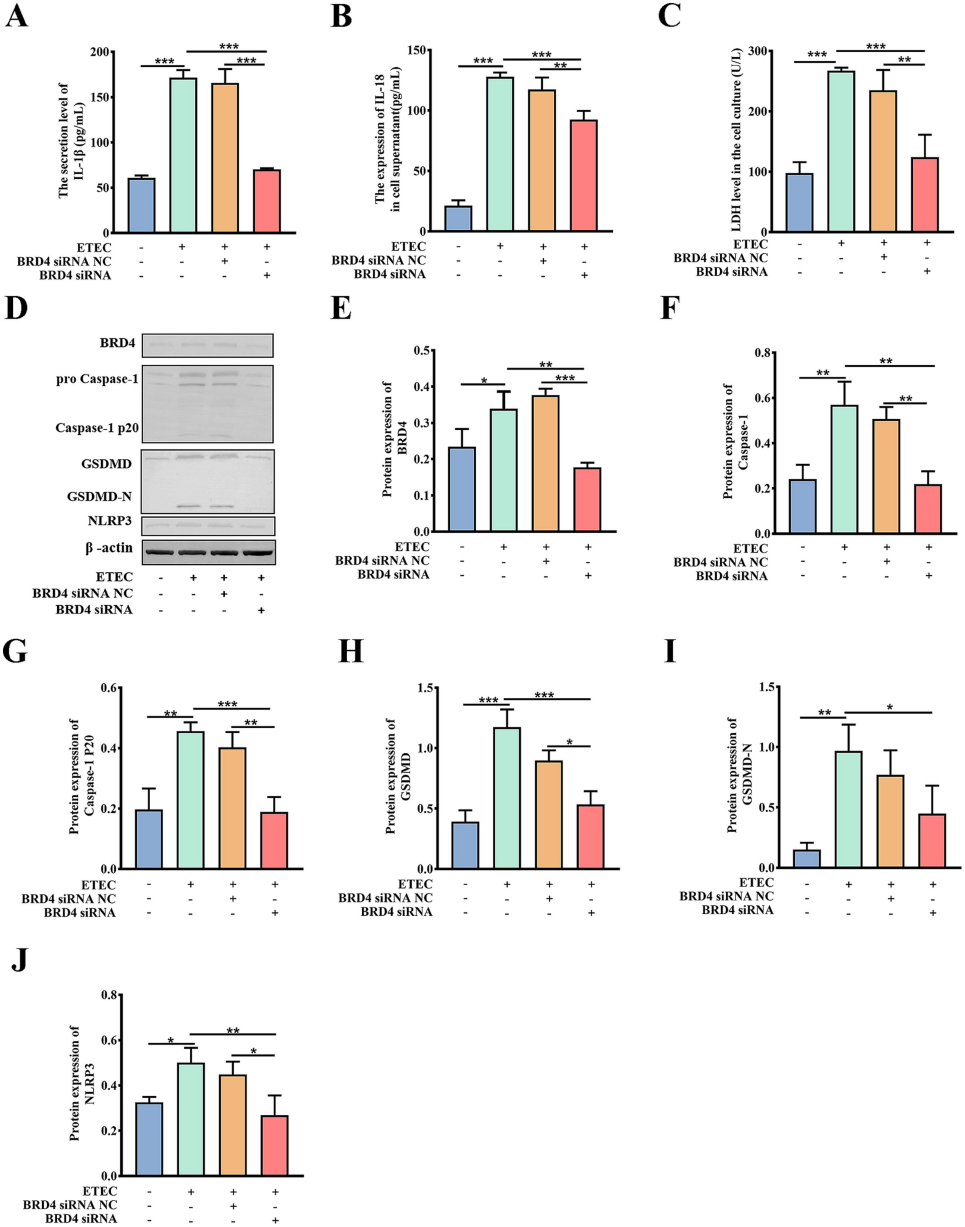
Biochemical characterization of pyroptotic indicators in BRD4 KD cells. IL-1β secretion **(A)**, IL-18 secretion **(B)**, and LDH release **(C)** of wild-type IPEC-J2 cells, ETEC-infected IPEC-J2 cells, ETEC-infected IPEC-J2 cells transfected with BRD4 siRNA NC (scrambled control), and ETEC-infected IPEC-J2 cells transfected with BRD4 siRNA. Protein expressions of pyroptosis-related indicators were detected by Western blot **(D)**, The relative protein levels of BRD4 **(E)**, Caspase-1 **(F)**, Caspase-1 P20 **(G)**, GSDMD **(H)**, GSDMD-N **(I)**, NLRP3 **(J)** were further quantitatively analyzed by ImageJ. *p ≤ 0.05, **p ≤ 0.01, ***p ≤ 0.001.

In order to validate that downstream BRD4 is involved in the biological function of the circ_0020647/ ssc-miR-185 network, we explored whether the silencing of BRD4 also affects the pyroptosis induced by ETEC infection. TEM photographs showed that the knockdown of BRD4 rescued the pyroptotic morphological characters, such as membrane pores and mitochondrial abnormalities in ETEC-infected cells (Figures 8A–D). We further confirmed that the proportion of caspase-1-mediated cell death was down-regulated in BRD4 KD cells post ETEC infection compared with the control treatments using flow cytometry (Figures 8E–I). As expected, the knockdown of BRD4 significantly reduced the secretion of cytokines IL-1β and IL-18 in ETEC-induced cells compared with the control group, indicating that BRD4 promoted inflammation stimulated by ETEC (Figures 9A,B). Next, the LDH release of different treatments was measured, resulting in consistent results that BRD4 was involved in promoting cell permeability induced by ETEC infection (Figure 9C). In addition, qRT-PCR (Supplementary Figure 3) and western blot (Figures 9D–J) analyses validated that mRNA and protein levels of pyroptosis indicators, including GADMD and caspase-1, were remarkably reduced when BRD4 was interfered with siRNA. Taken together, circ_0020647/SSC promoted ETEC-induced pyroptosis. Meanwhile, BRD4, which is the direct target of ssc-miR-185, also played a similar role in affecting pyroptosis. Therefore, our study reveals the mechanism of the circ_0020647/SSC/BRD4 signaling pathway in promoting the pyroptosis of IPEC-J2 cells induced by ETEC infection.

## 4 Discussion

**Figure 10:**
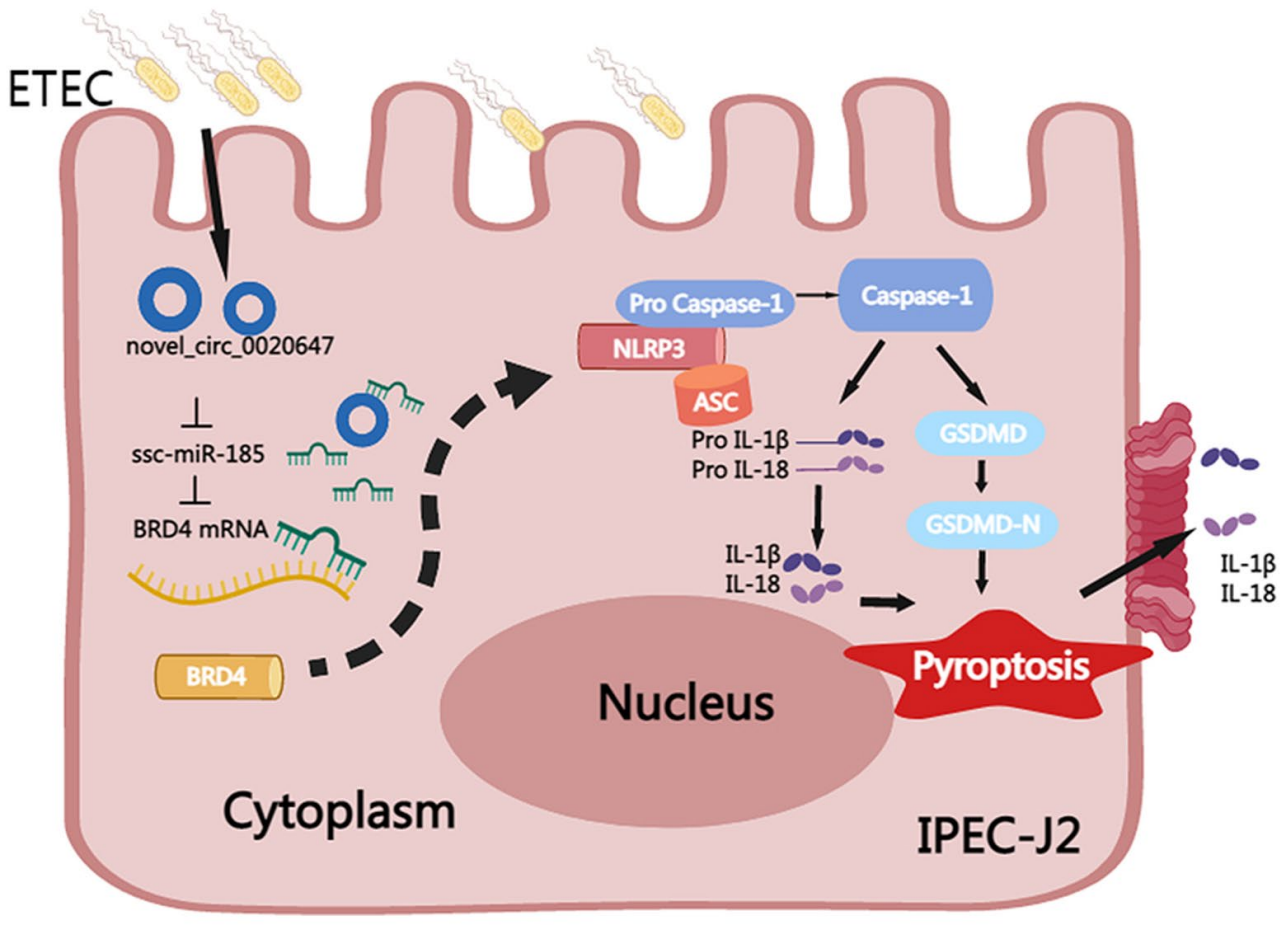
Mechanism of circ_0020647/ssc-miR-185/BRD4 ceRNA network in promoting pyroptosis of ETEC-infected IPEC-J2 cells. ETEC infection-induced up-regulation of circ_0020647 (blue circle), which further alleviates the inhibition of BRD4 (yellow) by sponging ssc-miR-185 (green). Increased BRD4 activates the NLRP3 inflammasome, which further leads to the activation of Caspase-1, maturation of IL-1β and IL-18, and cleavage of GSDMD. GSDMD-N thereby executes the secretion of IL-1β and IL-18, as well as the formation of membrane pores that result in pyroptosis.

In this study, the circRNA expression profile in ETEC-infected IPEC-J2 cells was characterized by high-throughput sequencing. The parental genes of DEcircRNAs were found to be extensively involved in immunity, cell proliferation and apoptosis-associated biological pathways, suggesting that circRNAs play an important regulatory role in the ETEC infection process. We identified a novel circRNA named circ_0020647 sponges ssc-miR-185 with further targets BRD4, promoting pyroptosis of IPEC-J2 cells induced by ETEC infection (Figure 10). We elucidated the regulatory role of the novel circ_0020647/ ssc-miR-185/ BRD4 ceRNA network in the pyroptosis of IPEC-J2 cells infected with ETEC, providing a theoretical basis for a better understanding of the mechanism of ETEC-induced intestinal barrier damage.

In recent years, the application of RNA sequencing technology has identified various classes of circRNAs that can originate from protein-coding or non-coding regions on the genome across different species. In the current study, we focused on circ_0020647, which is significantly differentially expressed after ETEC infection. Interestingly, circ_0020647 is transcribed from intergenic regions on the *sus scrofa* scaffold ID AEMK02000292.1. CircRNAs preferentially originate from precursor mRNAs via a back-splicing event, which is based on three known mechanisms including lariat-driven circularization, intron pairing-driven circularization, and RNA binding protein-driven circularization, resulting in the production of intronic circRNAs, exonic circRNAs and exon-intron circRNAs (16). By contrast, as a special class of circRNAs, intergenic circRNAs are generated from the intergenic genomic locus, containing two intronic segments flanked by GT-AG (or CT-AC) nucleotides as the splicing donor and acceptor sites for the looping junction. The biogenesis of intergenic circRNAs is reported to be related to pathological processes such as cancer and infectious diseases (17). In the present study, we identified a total of 3,075 intergenic circRNAs, which account for 3.97% of total circRNAs in IPEC-J2 cells. After ETEC infection, 18.18% of upregulated circRNAs and 9.05% of downregulated circRNAs are intergenic circRNAs, implicating that intergenic circRNAs may play an important role in regulating ETEC infection. Probably due to different sequencing platforms, 20.92% of the circRNAs were identified as intergenic in IPEC-J2 cells by Chen et al., whereas they did not show the number of intergenic circRNAs that were dysregulated due to porcine endemic diarrhea virus infection (18). Another study compared two breeds of pigs, among 275 DEcirRNAs discovered in porcine subcutaneous adipose tissue, 38 originated intergenically (19). The above studies showed that porcine intergenic circRNAs might be involved in regulating lipid metabolism, adipose deposition, or host defense against viral and bacterial infections.

Host circRNAs have been shown to play an important regulatory role in the pathogenesis of ETEC-induced diarrhea. Chen et al. identified 44 circRNAs in the lamb’s jejunum as potential biomarkers for ETEC F17 subtype-induced diarrhea, and their parental genes were enriched in intestinal inflammation-associated KEGG pathways (20). Zhao et al. analyzed the expression profiles of circRNAs in the intestinal cells of piglets susceptible or resistant to ETEC-F4ac subtype infection, and they found the parental genes of the DEcircRNAs were enriched in cytoskeleton, protein phosphorylation, cell adhesion, and ion transport pathways (15). Particularly, they emphasized that the enriched gap junction and adherens junction pathways are mainly associated with ETEC adhesion. Our KEGG pathway enrichment analysis also showed that the host genes of DEcircRNAs involved in ETEC infection were intensively enriched in immune responses and host-pathogen interaction-related pathways. Among them, the adherence junction and endocytosis pathways might be related to ETEC adherence and colonization, as well as enterotoxin delivery into host cells (21). Fc-epsilon-RI pathway, an immunoglobulin E (IgE) receptor, was found to mediate immune activation in the gastrointestinal tract, indicating an association with mucosal inflammation (22). The ubiquitin-mediated protein hydrolysis pathway mainly affects cell cycle progression, in which inefficient protein hydrolysis causes cell cycle dysregulation, leading to tumorigenesis (23). Thus, others and our study provide evidence that porcine circRNAs are involved in ETEC infection-induced gut inflammation development and enterocyte cell cycle abnormalities.

Competing endogenous RNAs (ceRNAs) are defined as coding or noncoding RNAs that can bind with miRNAs with complementary pairing, thus competing with the miRNA target transcripts for miRNA sequestration, leading to the block of miRNA inhibition on target transcripts. CircRNAs have been increasingly found to play their role via ceRNA activities. Zhao et al. created a comprehensive ceRNA network including 12 circRNAs, 10 miRNAs and 107 DE genes via bioinformatical analysis. Among them, they found circ-SORBS1 sponges ssc-miR-345-3p to regulate the expression of its parental gene SORBS1, which may further promote ETEC adhesion, indicating ceRNA networks may influence ETEC and host interaction (15). Lots of studies on human diseases have shown that circRNAs impact cell proliferation and death. In liver cancer cells, circMTO1 bound miR-9 to regulate p21 gene expression, resulting in tumor proliferation (24). Furthermore, the circRNA_0092516/miR-337-3p ceRNA network was found to regulate chondrocyte apoptosis in osteoarthritis (25). Our work demonstrated the involvement of circ_0020647 in targeting the ssc-miR-185/BRD4 axis to promote host cell pyroptosis during ETEC infection. Tremendous cirRNAs function as ceRNAs to compete for miRNA binding sites, thus regulating host cell viability, proliferation and other biological functions in many diseases.

Pyroptosis is a type of inflammatory cell death that can be triggered by various pathogens. The host and pathogens may compete to regulate pyroptosis to control the process of infection (26). ETEC K88 infection causes IPEC-1 cell inflammation and pyroptosis, while protocatechuic acid and quercetin treatment could alleviate ETEC K88-induced diarrhea by inhibiting the pyroptosis signaling pathway (10). We also observed the activation of NLRP3- Caspase-1-GSDMD pyroptosis pathway in ETEC F41-infected IPEC-J2 cells, confirming that ETEC-induced pyroptosis may promote diarrhea by disrupting the intestinal structure and barrier function. According to previous reports, pyroptosis occurred in intestinal epithelium during LPS-induced acute lung injury, while GSDMD deficiency alleviated intestinal mucosal injury, suggesting the role of pyroptosis in affecting intestinal mucosal barrier integrity (27). The viral pyroptosis mechanism remains controversial. Transmissible gastroenteritis virus (TGEV) infection resulted in NLRP3-dependent pyroptosis in porcine intestinal epithelial cells, indicating viral infection promoted host immune responses along with pyroptosis (28). While pyroptosis not only facilitates the elimination of infected host cells but also restricts intracellular virus viability. In this view, pyroptosis plays an essential role in maintaining the balance between host homeostasis and virus invasion. It has been reported that SARS-CoV-2 has evolved unclear mechanisms to inhibit host pyroptosis by its nucleocapsid protein via a caspase-1-dependent pathway, for the benefit of its own survival (29). However, the mechanisms of pyroptosis activation and inhibition balancing during pathogen infection need to be further investigated.

When pyroptosis occurs, the gasdermin family members, such as GSDMA/B/C/D/E, are cleaved into two fragments including the N-terminal fragment and the C-terminal fragment by the protease caspases. The N-terminal fragment which forms pores in the cell membrane, will finally lead to pyroptotic death and inflammatory cytokines (30). The assembly of NLRP3 inflammasomes can activate caspase-1 to promote GSDMD-mediated pyroptosis (7). Here we represented that circ_0020647 influences NLRP3/GSDMD/caspase-1 mediated pyroptosis of IPEC-J2 cells during ETEC infection. It has been reported that ETEC infection activates apoptosis via caspase-3 and caspase-8 dependent pathways in piglet jejunum (31). Apparently, the caspase family was highly activated by ETEC infection to participate in different types of cell death and immune responses. BRD4 has also been shown to be involved in the activation of NLRP3 inflammasome-mediated pyroptosis through the NF-κB signaling pathway (32). Inhibiting BRD4 interrupted the production of pro-inflammatory cytokines such as IL1β and IL18, and pyroptosis in acute colon injury induced by lipopolysaccharides (LPS), which is the essential glycolipid of Gram-negative bacteria like *E. coli* (33). This is consistent with the current study that BRD4 is an indicator of ETEC-induced pyroptosis and immune responses of porcine intestinal cells. Liu et al. identified BRD4 as a target gene of miR-218 in chronic obstructive pulmonary disease (COPD), and BRD4 transcription could be inhibited by overexpressing miR-218, resulting in the reduction of inflammation development (34). We further identified that the expression of BRD4 could be regulated by upstream circ_0020647/miR ssc-miR-185 ceRNA activities, providing clues for the complicated noncoding RNA regulatory networks on BRD4 expression in immune responses.

## 5 Conclusion

We successfully constructed a ceRNA network for the newly identified circRNA circ_0020647. Circ_0020647 sponges SSC-miR-185 which further targeted BRD4 to promote ETEC infection-induced IPEC-J2 cell pyroptosis via NLRP3/Caspase-1/GSDMD pyroptosis pathway (Figure 8). The present study suggests that the circ_0020647/ ssc-miR-185/ BRD4 ceRNA network may be a potential biomarker or therapeutic target for ETEC infection-induced piglet diarrhea.

## Data Availability

The datasets for this study can be found in the NCBI database with BioProject number PRJNA1068270.

## References

[1] Fleckenstein JM Kuhlmann FM. Enterotoxigenic *Escherichia coli* infections. Curr Infect Dis Rep. (2019) 21:9. doi: 10.1007/s11908-019-0665-x, PMID: 30830466 PMC6508951

[2] Dubreuil JD. Pig vaccination strategies based on enterotoxigenic *Escherichia coli* toxins. Braz J Microbiol. (2021) 52:2499–509. doi: 10.1007/s42770-021-00567-3, PMID: 34244980 PMC8270777

[3] Zhang Y Tan P Zhao Y Ma X. Enterotoxigenic *Escherichia coli*: intestinal pathogenesis mechanisms and colonization resistance by gut microbiota. Gut Microbes. (2022) 14:2055943. doi: 10.1080/19490976.2022.2055943, PMID: 35358002 PMC8973357

[4] Bin P Tang Z Liu S Chen S Xia Y Liu J. Intestinal microbiota mediates Enterotoxigenic *Escherichia coli*-induced diarrhea in piglets. BMC Vet Res. (2018) 14:385. doi: 10.1186/s12917-018-1704-9, PMID: 30518356 PMC6282381

[5] Qiao J Sun Z Liang D Li H. *Lactobacillus salivarius* alleviates inflammation via NF-κB signaling in ETEC K88-induced IPEC-J2 cells. J Anim Sci Biotechnol. (2020) 11:76. doi: 10.1186/s40104-020-00488-5, PMID: 32774852 PMC7398071

[6] Tian M Li L Tian Z Zhao H Chen F Guan W. Glyceryl butyrate attenuates enterotoxigenic *Escherichia coli*-induced intestinal inflammation in piglets by inhibiting the NF-κB/MAPK pathways and modulating the gut microbiota. Food Funct. (2022) 13:6282–92. doi: 10.1039/d2fo01056a, PMID: 35607985

[7] Xia Y Bin P Liu S Chen S Yin J Liu G. Enterotoxigenic *Escherichia coli* infection promotes apoptosis in piglets. Microb Pathog. (2018) 125:290–4. doi: 10.1016/j.micpath.2018.09.032, PMID: 30243552

[8] Galluzzi L Vitale I Aaronson SA Abrams JM Adam D Agostinis P. Molecular mechanisms of cell death: recommendations of the nomenclature committee on cell death 2018. Cell Death Differ. (2018) 25:486–541. doi: 10.1038/s41418-017-0012-4, PMID: 29362479 PMC5864239

[9] Zhang KJ Wu Q Jiang SM Ding L Liu CX Xu M. Pyroptosis: a new frontier in kidney diseases. Oxidative Med Cell Longev. (2021) 2021:6686617. doi: 10.1155/2021/6686617, PMID: 34007404 PMC8102120

[10] Xiao K Zhou M Lv Q He P Qin X Wang D. Protocatechuic acid and quercetin attenuate ETEC-caused IPEC-1 cell inflammation and injury associated with inhibition of necroptosis and pyroptosis signaling pathways. J Anim Sci Biotechnol. (2023) 14:5. doi: 10.1186/s40104-022-00816-x, PMID: 36721159 PMC9890695

[11] Kristensen LS Andersen MS Stagsted LVW Ebbesen KK Hansen TB Kjems J. The biogenesis, biology and characterization of circular RNAs. Nat Rev Genet. (2019) 20:675–91. doi: 10.1038/s41576-019-0158-7, PMID: 31395983

[12] Yue B Wang J Ru W Wu J Cao X Yang H. The circular RNA circHUWE1 sponges the miR-29b-AKT3 Axis to regulate myoblast development. Mol Ther Nucleic Acids. (2020) 19:1086–97. doi: 10.1016/j.omtn.2019.12.039, PMID: 32045877 PMC7015828

[13] Liu J Liu Y Zhang L Chen Y Du H Wen Z. Down-regulation of circDMNT3B is conducive to intestinal mucosal permeability dysfunction of rats with sepsis via sponging miR-20b-5p. J Cell Mol Med. (2020) 24:6731–40. doi: 10.1111/jcmm.15324, PMID: 32383354 PMC7299677

[14] Li ZX Wang LX Zhang Y Chen W Zeng YQ. circGLI3 inhibits oxidative stress by regulating the miR-339-5p/VEGFA Axis in IPEC-J2 cells. Biomed Res Int. (2021) 2021:1086206. doi: 10.1155/2021/1086206, PMID: 34423029 PMC8376464

[15] Zhao Q Xu Q Serafino MA Zhang Q Wang C Yu Y. Comprehensive analysis of circular RNAs in porcine small intestine epithelial cells associated with susceptibility to *Escherichia coli* F4ac diarrhea. BMC Genomics. (2023) 24:211. doi: 10.1186/s12864-022-08994-8, PMID: 37085748 PMC10122348

[16] Misir S Wu N Yang BB. Specific expression and functions of circular RNAs. Cell Death Differ. (2022) 29:481–91. doi: 10.1038/s41418-022-00948-7, PMID: 35169296 PMC8901656

[17] Pisignano G Michael DC Visal TH Pirlog R Ladomery M Calin GA. Going circular: history, present, and future of circRNAs in cancer. Oncogene. (2023) 42:2783–800. doi: 10.1038/s41388-023-02780-w, PMID: 37587333 PMC10504067

[18] Chen J Wang H Jin L Wang L Huang X Chen W. Profile analysis of circRNAs induced by porcine endemic diarrhea virus infection in porcine intestinal epithelial cells. Virology. (2019) 527:169–79. doi: 10.1016/j.virol.2018.11.014, PMID: 30530223 PMC7112103

[19] Li A Huang W Zhang X Xie L Miao X. Identification and characterization of CircRNAs of two pig breeds as a new biomarker in metabolism-related diseases. Cell Physiol Biochem. (2018) 47:2458–70. doi: 10.1159/000491619, PMID: 29990990

[20] Chen W Lv X Zhang W Hu T Cao X Ren Z. Non-coding transcriptome provides novel insights into the *Escherichia coli* F17 susceptibility of sheep lamb. Biology. (2022) 11:348. doi: 10.3390/biology11030348, PMID: 35336723 PMC8945857

[21] Kesty NC Mason KM Reedy M Miller SE Kuehn MJ. Enterotoxigenic *Escherichia coli* vesicles target toxin delivery into mammalian cells. EMBO J. (2004) 23:4538–49. doi: 10.1038/sj.emboj.7600471, PMID: 15549136 PMC533055

[22] Bannert C Bidmon-Fliegenschnee B Stary G Hotzy F Stift J Nurko S. Fc-epsilon-RI, the high affinity IgE-receptor, is robustly expressed in the upper gastrointestinal tract and modulated by mucosal inflammation. PLoS One. (2012) 7:e42066. doi: 10.1371/journal.pone.0042066, PMID: 22848703 PMC3407106

[23] Dang F Nie L Wei W. Ubiquitin signaling in cell cycle control and tumorigenesis. Cell Death Differ. (2021) 28:427–38. doi: 10.1038/s41418-020-00648-0, PMID: 33130827 PMC7862229

[24] Han D Li J Wang H Su X Hou J Gu Y. Circular RNA circMTO1 acts as the sponge of microRNA-9 to suppress hepatocellular carcinoma progression. Hepatology. (2017) 66:1151–64. doi: 10.1002/hep.29270, PMID: 28520103

[25] Huang Z Ma W Xiao J Dai X Ling W. CircRNA_0092516 regulates chondrocyte proliferation and apoptosis in osteoarthritis through the miR-337-3p/PTEN axis. J Biochem. (2021) 169:467–75. doi: 10.1093/jb/mvaa119, PMID: 33135071

[26] Bergsbaken T Fink SL Cookson BT. Pyroptosis: host cell death and inflammation. Nat Rev Microbiol. (2009) 7:99–109. doi: 10.1038/nrmicro2070, PMID: 19148178 PMC2910423

[27] Zhao J Wang H Zhang J Ou F Wang J Liu T. Disulfiram alleviates acute lung injury and related intestinal mucosal barrier impairment by targeting GSDMD-dependent pyroptosis. J Inflamm. (2022) 19:17. doi: 10.1186/s12950-022-00313-y, PMID: 36266722 PMC9582395

[28] Wei G Luo S Wu W Hu J Zhou R. Activation of interleukin-1*β* release and Pyroptosis by transmissible gastroenteritis virus is dependent on the NOD-like receptor protein 3 Inflammasome in porcine intestinal epithelial cell line. Viral Immunol (2021) 34(6):401–409. doi: 10.1089/vim.2020.0227, PMID: 33973805

[29] Ma J Zhu F Zhao M Shao F Yu D Ma J. SARS-CoV-2 nucleocapsid suppresses host pyroptosis by blocking Gasdermin D cleavage. EMBO J. (2021) 40:e108249. doi: 10.15252/embj.2021108249, PMID: 34296442 PMC8420271

[30] Ding J Wang K Liu W She Y Sun Q Shi J. Pore-forming activity and structural autoinhibition of the gasdermin family. Nature. (2016) 535:111–6. doi: 10.1038/nature18590, PMID: 27281216

[31] Zhaolin Z Guohua L Shiyuan W Zuo W. Role of pyroptosis in cardiovascular disease. Cell Prolif. (2019) 52:e12563. doi: 10.1111/cpr.12563, PMID: 30525268 PMC6496801

[32] Hua T Wang H Fan X An N Li J Song H. BRD4 inhibition attenuates inflammatory pain by ameliorating NLRP3 Inflammasome-induced Pyroptosis. Front Immunol. (2022) 13:837977. doi: 10.3389/fimmu.2022.837977, PMID: 35154163 PMC8826720

[33] Chen L Zhong X Cao W Mao M Li W Yang H. JQ1 as a BRD4 inhibitor blocks inflammatory Pyroptosis-related acute Colon injury induced by LPS. Front Immunol. (2021) 12:609319. doi: 10.3389/fimmu.2021.609319, PMID: 33679744 PMC7930386

[34] Liu X Wang J Luo H Xu C Chen X Zhang R. MiR-218 inhibits CSE-induced apoptosis and inflammation in BEAS-2B by targeting BRD4. Int J Chron Obstruct Pulmon Dis. (2020) 15:3407–16. doi: 10.2147/COPD.S278553, PMID: 33408470 PMC7781039

