# Supplementary material for: A novel circular RNA circ_0020647 promotes ETEC-induced IPEC-J2 cell pyroptosis via the ssc-miR-185/BRD4 axis
Source: Front Vet Sci. 2025 Jun 4;12:1578941. doi: 10.3389/fvets.2025.1578941 (PMC12175092; doi:10.3389/fvets.2025.1578941)
Supplement: Supplementary file 1 [file Data_Sheet_1.pdf]

Supplementary Material

1 Supplementary Figures and Tables

1.1 Supplementary Figures

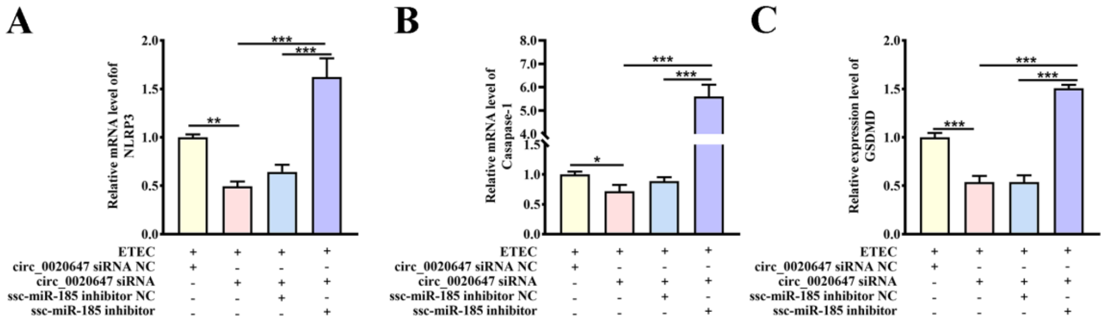

Supplementary Figure 1. Relative mRNA expression of NLRP3 **(A)**, Caspase-1 **(B)**, GSDMD **(C)** detected by qRT-PCR.

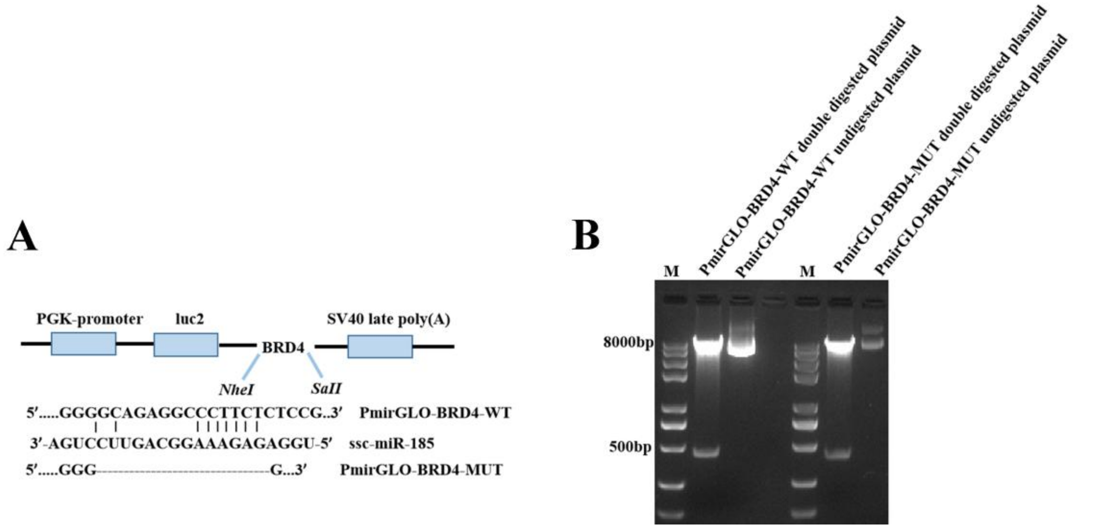

Supplementary Figure 2. The construction of PmirGLO-BRD4-WT/MUT plasmids. **(A)** PmirGLO-BRD4-WT/MUT plasmid construction schematic diagram, and the prediction of BRD4 3'UTR and ssc-miR-185 binding site. **(B)** Electrophoresis of PmirGLO-BRD4-WT (lane 1: double digested plasmid; lane 2: undigested plasmid) and PmirGLO-BRD4-MUT (lane 3: double digested plasmid; lane 4: undigested plasmid) digested by NheI and SalI.

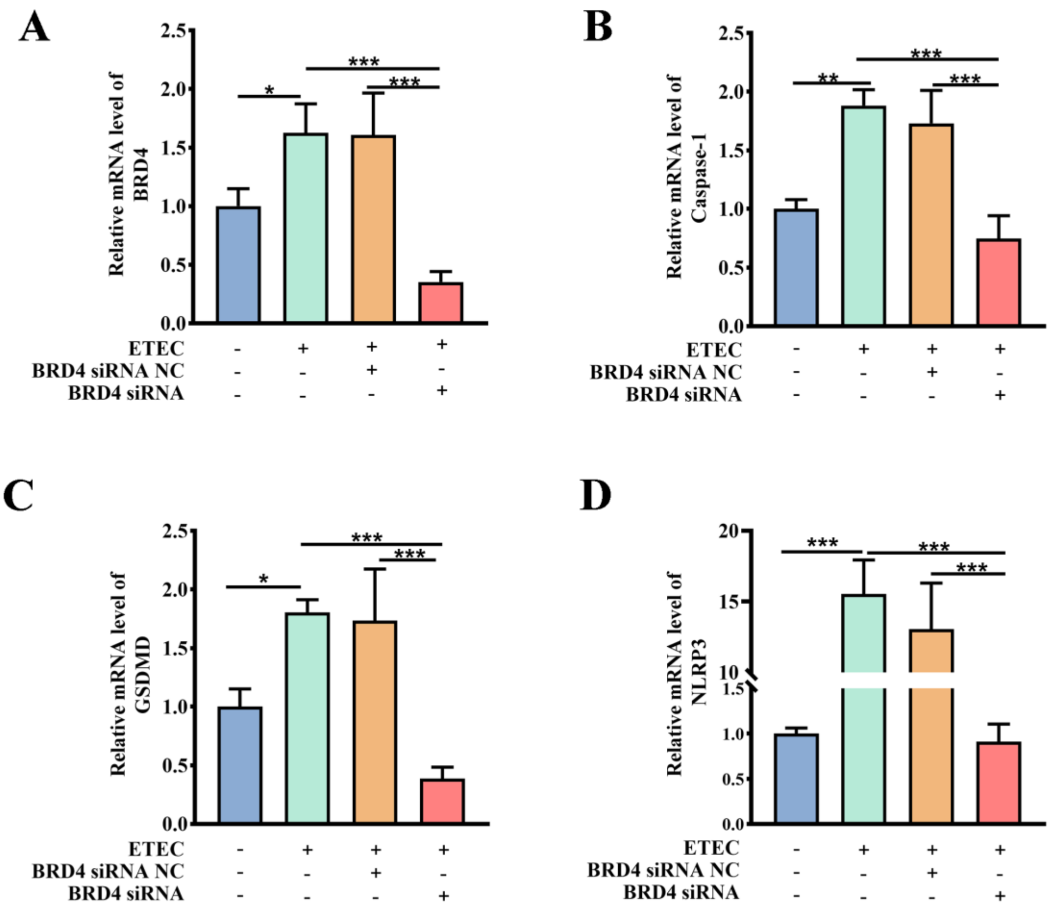

Supplementary Figure 3. Relative mRNA expression of BRD4 **(A)**, Caspase-1 **(B)**, GSDMD **(C)**, NLRP3 **(D)** detected by qRT-PCR.
